# Supplementary material for: Polydopamine-Coated Laponite Nanoplatforms for Photoacoustic Imaging-Guided Chemo-Phototherapy of Breast Cancer
Source: Nanomaterials (Basel). 2021 Feb 4;11(2):394. doi: 10.3390/nano11020394 (PMC7913843; doi:10.3390/nano11020394)
Supplement: Supplementary file 1 [file nanomaterials-11-00394-s001.pdf]

nanomaterials

Supplementary Materials

Polydopamine-Coated Laponite Nanoplatforms for Photoacoustic Imaging-Guided Chemo-Phototherapy of Breast Cancer

Renna Liu [1], Fanli Xu [1], Lu Wang [1], Mengxue Liu [1], Xueyan Cao [1], Xiangyang Shi [1,2,*] and Rui Guo [1,2,*]

[1] College of Chemistry, Chemical Engineering and Biotechnology, Donghua University, Shanghai 201620, China; renna_liu906@163.com (R.L.); xufanli1992@163.com (F.X.); wanglu2013@dhu.edu.cn (L.W.); 13262579590@163.com (M.L.); caoxy_116@dhu.edu.cn (X.C.)

[2] State Key Laboratory for Modification of Chemical Fibers and Polymer Materials, College of Materials Science and Engineering, Donghua University, Shanghai 201620, China

* Correspondence: xshi@dhu.edu.cn (X.S.); ruiguo@dhu.edu.cn (R.G.). Tel.: +86-21-6779-2750 (R.G.); Fax: +86-21-67-792-306-804 (R.G.)

Part of Experimental Section

Materials

Laponite ($Na^{+}_{0.7}[(Si_8Mg_{5.5}Li_{0.3})O_{20}(OH)_4]^{-}_{0.7}$, LAP) was purchased from Guangzhou Bo Feng Chemical Technology Co., Ltd. (Guangzhou, China). Indocyanine Green ($C_{43}H_{47}N_2NaO_6S_2$ > 90%, ICG), dopamine ($C_8H_{11}NO_2$·HCl > 98%, DA), ethanol (C_2H_5OH) and ammonium hydroxide (NH_4OH) were purchased from J&K Chemical Ltd. (Shanghai, China). *m*PEG-NH_2 and NH_2-PEG-Mal (M_w = 5000) were obtained from Shanghai Yarebio. Co., Ltd. (Shanghai, China). Thiolated cyclic RGD peptide (M_w = 690.93) was purchased from GenicBio (Shanghai, China). Cell Counting Kit-8 (CCK-8) was obtained from Beyotime Biotechnology (Shanghai, China). All chemicals were used without further purification. 4T1 cells (murine breast carcinoma cell lines) and MCF-7 cells (human breast adenocarcinoma cell lines) were acquired from Chinese Academy of Sciences cell bank (Shanghai, China). Dulbecco's modified eagle medium (DMEM), penicillin, streptomycin, and fetal bovine serum (FBS) were purchased from Hangzhou Jinuo Biomedical Technology (Hangzhou, China). Water used in all experiments was purified using a Milli-Q Plus. 185 water purification system (Millipore, Bedford, MA, USA) with a resistivity higher than 18.2 MΩ·cm. Microsep with a molecular weight cut-off of 10,000 was purchased from Merck Millipore Ltd., (Kenilworth, NJ, USA). Two type of 808 nm laser (FC-808-10W-MM with a beam spot size of 2.5 cm^2 and DL-808-2000-T with a beam spot size of 0.25 cm^2) were purchased from Shanghai Xilong Optoelectronics Technology Co. Ltd., (Shanghai, China).

Characterization Techniques

UV-Vis spectroscopy was obtained using a Lambda 25 UV-Vis spectrophotometer (Perkin-Elmer, Waltham, MA, USA). ICG/LAP-PDA-PEG-RGD/DOX NPs were observed by SEM (JEOL JSM-5600LV, Akishima City, Japan). The samples were sputter-coated with gold films before SEM observation. And the diameters of NPs were analyzed by at least 200 NPs from different images using ImageJ 1.40G software (http://rsb.info.nih.gov/ij/download.html, National Institutes of Health, USA). Zeta potential and dynamic light scattering (DLS) measurements were carried out using a Malvern Zetasizer Nano ZS model ZEN3600 (Worcestershire, U.K.) equipped with a standard 633 nm laser. Samples were dissolved in different aqueous media (H_2O, normal saline, PBS and DMEM) with a concentration of 1 mg/mL before analysis. The Si concentrations of samples were analyzed by using a Leeman Prodigy ICP-OES system (Hudson, NH, USA). The samples were digested by aqua regia and diluted with water before measurements.

The Photothermal and PA imaging property of ICG/LAP-PDA-PEG-RGD solution

For photothermal conversion measurement, ICG, LAP, ICG/LAP-PDA-*m*PEG, and ICG/LAP-PDA-PEG-RGD solutions at the ICG concentration of 100 μg/mL were stored in Eppendorf tubes, and then irradiated by an 808 nm laser (0.25 cm^2, 1.2 W/cm^2, 3 min) (Shanghai Xilong Optoelectronics Technology Co. Ltd., Shanghai, China). During irradiation, the probe was placed in the solution to record the temperature changes every 5 s by an online DT-8891 Ethermocouple thermometer (Shenzhen Everbest Machinery Industry Co., Ltd., Shenzhen, China). For PA imaging measurement, ICG/LAP-PDA-*m*PEG and ICG/LAP-PDA-PEG-RGD solutions with different ICG concentrations (0, 90, 120 and 150 μg/mL) were scanned by the Vevo LAZR PA imaging system (Visualsonics Inc., Toronto, ON, Canada) [1,2]. And the intensity of PA signal was evaluated in arbitrary units (a.u.) by selecting a region of interest in the digital PA images.

In vitro release assay

The DOX encapsulation efficiency can be calculated by measuring the concentration of free DOX in the collected filtrate after 3 times of centrifugation using a Lambda 25 UV-vis spectrophotometer (PerkinElmer, Waltham, MA, USA) at 480 nm and a standard DOX absorbance-concentration calibration curve.

ICG/LAP-PDA-PEG-RGD/DOX were dispersed in 1 mL of acetate buffer (pH = 5.0) or phosphate buffer (pH = 7.4) at a concentration of 1.4 mg/mL, and then sealed in a dialysis bag (cutoff Mw = 3500). The dialysis bag was immersed in 9 mL buffer solution (pH 5.0 or 7.4) and placed in shaker at 37°C for 24 h. After 1, 2, 4, 6, 8, 12 and 24 h, 1 mL of solution in the tube was taken out to measure the released amount of DOX (λ_{DOX} = 480 nm) by UV-vis spectrophotometer, and 1 mL of corresponding buffer solution was added. For laser irradiation group, the dialysis bag was irradiated by an 808 nm laser (1.2 W/cm^2) for 5 min at different time intervals, and the amount of DOX released was measured in the same way.

In vitro assays

4T1 cells (the murine breast cancer cell line) were regularly cultured in DMEM medium supplemented with 10% Fetal Bovine Serum (FBS), 100 U/mL penicillin, and 100 U/mL streptomycin at 37 °C in a 5% CO_2 incubator.

4T1 cells were seeded in 96-well plate with a density of 0.8 × 10^4 cells per well. When the cells were completely adhered, ICG/LAP-PDA-PEG-RGD medium solution at different concentrations (12.5, 25, 50, 75, and 100 μg/mL) were added. After being incubated for 24 h, the medium was poured out and the cells were washed with PBS. Finally, the cells were incubated with 100 μL of serum free medium containing 10 μL of CCK-8 solution at 37°C for another 4 h in a dark environment. The absorption value at 450 nm was determined by a microplate reader (Multiskan MK3, Massachusetts, MA, USA).

In order to evaluate the inhibition effect of ICG/LAP-PDA-PEG-RGD/DOX, 4T1 cells (0.8 × 10^4 cells/well) were cultured in 96-well plate overnight. Then the medium containing PBS, DOX, ICG/LAP-PDA-PEG-RGD and ICG/LAP-PDA- PEG-RGD/DOX at different DOX concentrations (1.5, 3.0, 4.5, 6.0, 9.0 μg/mL) were added and cultured for 6 h. For laser irradiation group, the cells were irradiated by an 808 nm laser (1.2 W/cm^2) for 5 min after washed with saline for 3 times [3]. Finally, the absorbance values of different groups were determined by CCK-8 assay, and the cell survival rate was calculated.

4T1 cells were seeded in 12-well plates at a density of 2 × 10^5 cells per well and incubated at 37 °C and 5% CO_2 overnight. After that, the medium was substituted by DMEM containing ICG/LAP-PDA-*m*PEG or ICG/LAP-PDA-PEG-RGD at different ICG concentrations (0, 2.5, 5.0, 10, 20 and 40 μg/mL). After 6 h incubation, the cells were washed with PBS 3 times, trypsinized, resuspended, counted and lysed using an aqua regia solution (nitric acid/hydrochloric acid, v/v = 3:1). The cellular uptake of the Si element was measured by ICP-OES. For RGD blocking group, 4T1 cells were pre-incubated with 5 μM free RGD peptide overnight before treatment with ICG/LAP-PDA-PEG-RGD. MCF-7 cells

were used as $\alpha_v\beta_3$ negative control cell line to evaluate the targeting efficacy of ICG/LAP-PDA-PEG-RGD by the similar method.

In vivo PA Imaging and biodistribution

All animal experiments were carried out according to the protocols approved by the ethical committee of Donghua University for animal care and also in accordance with the policy of the National Ministry of Health. BALB/c nude mice (5 weeks old, Shanghai Slac Laboratory Animal Center, Shanghai, China) were used to build up a mouse breast cancer tumor model. After feeding for one week, 4T1 cells (1×10^6 cells, in 100 μL PBS) were injected into the right hind leg of each mouse to generate a subcutaneous tumor. When the size of tumor reached a volume of 0.08–0.1 cm^3, the mice were anesthetized by intraperitoneal injection of pentobarbital sodium (40 mg kg^{-1}), and then intravenously injected with ICG/LAP-PDA-PEG-RGD and ICG/LAP-PDA-*m*PEG (0.1 mL PBS, 3 mg/mL). The image of tumor site was scanned (0.5, 1, 2 and 4 h) by Vevo LAZR photoacoustic imaging system equipped with an 875 nm laser (Visual Sonics Inc., Toronto, ON, Canada).

The tumor-bearing mice after PA scanning were euthanized at 2 h post-injection, and the heart, liver, spleen, lung, kidney, and tumor were extracted and weighted. The organs were cut into pieces and digested by aqua regia solution for 24 h. Then, the Si content in different organs was quantified by ICP-OES. For comparison, the mice injected with PBS were used as control.

In vivo antitumor therapeutic efficacy

The 4T1 tumor-bearing nude mice were set and assigned to 6 groups (n=5): PBS, PBS+L, ICG/LAP-PDA-PEG-RGD, ICG/LAP-PDA-PEG-RGD+L (phototherapy), ICG/LAP-PDA-PEG-RGD/DOX (chemotherapy), and ICG/LAP-PDA-PEG-RGD/ DOX+L (photo-chemotherapy). Same amount of nanoplatforms (100 μL, 3 mg/mL) were intratumorally injected into the tumor bearing nude mice on Day 0 and Day 3, and 10 min after administration, mice were irradiated with 808 nm NIR laser at a power of 1.2 W/cm^2 for 5 min. The tumor volume and body weight of all mice were recorded every other day, and the pictures of mice and tumors were taken by digital camera.

Histological examinations

After photothermal treatment, tumors and major organs were excised from euthanized mice, followed by hematoxylin and eosin (H&E) staining. Standard TdT mediated dUTP nick-end labeling (TUNEL) staining tests were performed according to our previous work to confirm the tumor cell apoptosis efficacy [2].

Statistical Analysis

One-way ANOVA statistical analysis was performed to evaluate the experimental data. A p value of 0.05 was selected as the significance level, and the data were indicated with (*) for $p < 0.05$, (**) for $p < 0.01$, and (***) for $p < 0.001$, respectively.

Table S1. Zeta potential and hydrodynamic size of ICG/LAP-PDA-*m*PEG, ICG/LAP-PDA-PEG-RGD and ICG/LAP-PDA-PEG-RGD/DOX, respectively.

Materials	Zeta potential (mV)	Hydrodynamic size (nm)	Polydispersity index (PDI)
ICG/LAP-PDA-*m*PEG	−29.5 ± 0.62	104.2 ± 4.76	0.315 ± 0.043
ICG/LAP-PDA-PEG-RGD	−27.8 ± 0.62	109.6 ± 1.15	0.404 ± 0.019
ICG/LAP-PDA-PEG-RGD/DOX	−17.1 ± 0.95	148.2 ± 3.10	0.236 ± 0.011

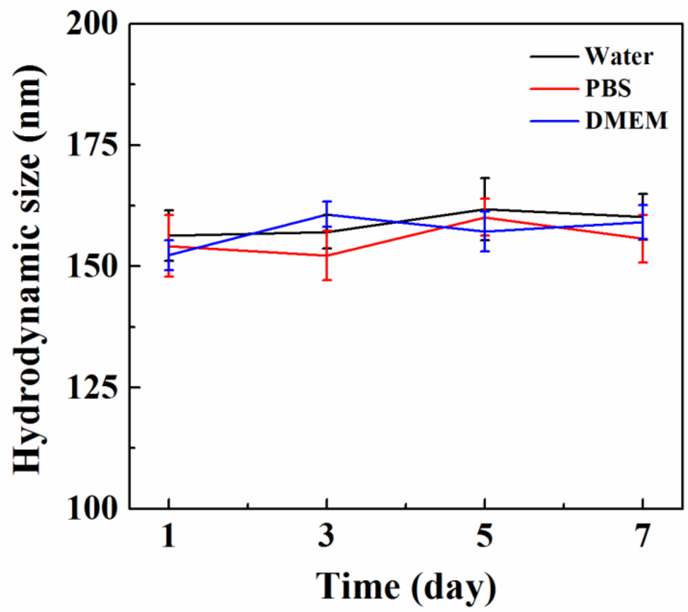

Figure S1. Hydrodynamic size of ICG/LAP-PDA-PEG-RGD/DOX dispersed in water, PBS and DMEM in 7 days.

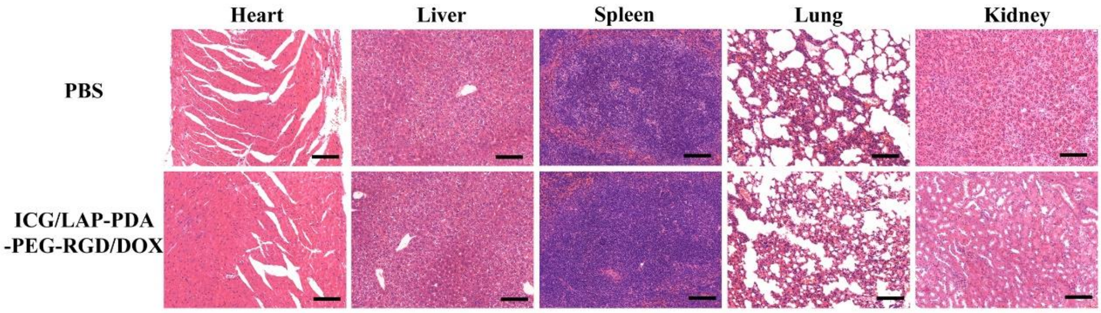

Figure S2. H&E staining section images of major organs from mice after PBS and ICG/LAP-PDA-PEG-RGD/DOX treatment (scale bar: 100 μm).

References

1. Zhang, C.C.; Sun, W.J.; Wang, Y.; Xu, F.; Qu, J.; Xia, J.D.; Shen, M.W.; Shi, X.Y., Gd-/CuS-Loaded Functional Nanogels for MR/PA Imaging-Guided Tumor-Targeted Photothermal Therapy. *ACS Appl. Mater. Interfaces* **2020,** *12*, 9107–9117.
2. Liu, M.; Zhang, J.; Li, X.; Cai, C.; Cao, X.; Shi, X.; Guo, R., A polydopamine-coated LAPONITE®-stabilized iron oxide nanoplatform for targeted multimodal imaging-guided photothermal cancer therapy. *J. Mater. Chem. B* **2019**, *7*, 3856–3864.
3. Xu, F.L.; Liu, M.X.; Li, X.; Xiong, Z.J.; Cao, X.Y.; Shi, X.Y.; Guo, R., Loading of Indocyanine Green within Polydopamine-Coated Laponite Nanodisks for Targeted Cancer Photothermal and Photodynamic Therapy. *Nanomaterials* **2018**, *8*, 347–360.
